# A drop in serum estradiol levels during GnRH antagonist cotreatment in cycles stimulated with gonadotropins is associated with lower cumulative live birth rates

**DOI:** 10.3389/fendo.2026.1722379

**Published:** 2026-03-18

**Authors:** Lara Janssens, Ella Roelant, Christophe Blockeel, Diane De Neubourg

**Affiliations:** 1 Department Center for Reproductive Medicine, Antwerp University Hospital, Edegem, Belgium; 2 Faculty of Medicine and Health Sciences, University of Antwerp, Brussel, Belgium; 3 Clinical Trial Center (CTC), CRC Antwerp, Antwerp University Hospital, University of Antwerp, Edegem, Belgium; 4 Brussels IVF, Centre for Reproductive Medicine, Brussels University Hospital, Brussel, Belgium

**Keywords:** assisted reproductive technology, estradiol, GnRH antagonist, *in vitro* fertilization, ovarian stimulation

## Abstract

Over the last decades, the gonadotropin-releasing hormone (GnRH) antagonist protocol has become widely used for prevention of premature luteinizing hormone surge during ovarian stimulation with exogenous gonadotropins. Literature has shown its efficacy and safety, while maintaining similar live birth rates compared to agonist protocols. Clinicians occasionally notice a drop in serum estradiol levels after GnRH antagonist initiation. This study aimed to analyze the impact of a drop in serum estradiol levels after the administration of a GnRH antagonist on clinical outcomes in *in vitro* fertilization (IVF)/intracytoplasmic sperm injection (ICSI) cycles. The results showed that estradiol drop was related to lower ongoing pregnancy, live birth and cumulative live birth rates. Estradiol drop was less frequent in cycles using gonadotropins containing luteinizing hormone (LH) activity and cycles with a drop showed a larger decrease in serum LH after the first GnRH antagonist administration, resulting in lower serum values on the day of ovulation triggering. These findings suggest that future prospective research could focus on potential optimization of luteinizing hormone (LH) levels and this study might also add to the discussion that estradiol monitoring could be useful in detecting cycles with an increased risk of less favorable outcomes.

## 1 Introduction

Infertility is a significant global issue, affecting around one in six couples worldwide (1) and high quality care is required. Assisted reproductive technologies (ART), such as *in vitro* fertilization (IVF), have progressed tremendously, with a total of 2.8 million ART cycles performed worldwide in 2016 (2).

Over the last decades, the gonadotropin-releasing hormone (GnRH) antagonist protocol has become widely used for prevention of premature luteinizing hormone (LH) surge during ovarian stimulation (OS) with exogenous gonadotropins (3). The latest ESHRE guideline on OS recommends the use of the antagonist protocol over the agonist protocol, given its comparable efficacy and higher safety (4, 5). Meta-analyses showed similar live birth rates in antagonist and long agonist protocols, with the antagonist protocol associated with a lower incidence of ovarian hyperstimulation syndrome (OHSS) and being more patient friendly due to its immediate pituitary suppression (6, 7).

In order to monitor ovarian response to the chosen treatment protocol, serum estradiol can support ultrasound findings and assess follicular growth and oocyte maturation (8). Literature on serum estradiol levels in GnRH antagonist cycles focuses mainly on the timing of initial antagonist administration and estradiol levels on the day of ovulation triggering (9–11). The timing of the initial administration depends on whether the patient receives a fixed or flexible antagonist protocol. In the fixed protocol, the GnRH antagonist is started on day five or six of OS. In a flexible protocol, antagonist administration is started when a dominant follicle reaches a diameter of at least 14 or 15 mm and/or serum estradiol levels surpass a threshold (6). There is no clear consensus yet on whether a fixed or flexible protocol is optimal (12). However, the meta-analysis of Al-Inany et al. found that starting antagonist administration when serum estradiol levels are below 300 pg/ml might be associated with an estradiol drop or impaired estradiol increase (6).

One phenomenon remains a gap in the literature. In cycles treated with an antagonist protocol, clinicians occasionally notice a drop in serum estradiol levels after antagonist initiation. In physiological conditions, theca cells produce androgens under the influence of LH and are converted to estrogens in the granulosa cells by aromatase driven by the action of follicle-stimulating hormone (FSH). However, administration of GnRH antagonists leads to immediate suppression of endogenous LH and FSH secretion (13). Because the patient administers FSH during ovarian stimulation, the conversion from androgens to estrogens is usually not a problem, but most gonadotropin preparations contain very little amounts of LH and/or LH activity and meanwhile endogenous LH levels can be very low throughout ovarian stimulation cycles. There has been very little research on the impact of GnRH antagonists on estradiol levels. The only research findings are observations along dose findings studies for GnRH antagonists that show that the higher the dose of the GnRH antagonist, the lower the serum LH and estradiol levels throughout the stimulation phase (14, 15). Because our previous research showed a significant decrease in serum LH in GnRH antagonist cycles, both with recombinant FSH and human menopausal gonadotropin (hMG) and because no decrease in serum LH in GnRH agonist long cycles was detected (16), we were triggered to examine the potential impact of the GnRH antagonist on estradiol levels throughout the ovarian stimulation cycle and its relation with LH.

Regular monitoring consisting of ultrasound and hormonal blood analysis allows us to observe this estradiol drop shortly after the initial antagonist administration. The ESHRE guideline on OS states that the addition of estradiol measurements to ultrasound monitoring is probably not recommended (4). However, it also mentions that this recommendation is based on a meta-analysis of six studies, of which four used exclusively GnRH agonists and two used both agonist and antagonist protocols (17). One could ask if this advice is valid in GnRH antagonist protocols.

This study analyzed clinical outcomes of cycles with an estradiol drop after GnRH antagonist initiation and the association between estradiol drop and cycle characteristics.

## 2 Materials and methods

### 2.1 Design and study population

This was a retrospective, non-interventional cohort study of patients receiving ART. Patients were treated at the Center for Reproductive Medicine of the Antwerp University Hospital. Women between 20 and 45 years of age were included, and they were treated for IVF/intracytoplasmic sperm injection (ICSI), preimplantation genetic testing (PGT) or oocyte vitrification for fertility preservation purposes. Exclusion criteria were oocyte vitrification for oncologic purposes due to potential confounders related to oncology treatments, the use of clomiphene citrate and the use of a GnRH agonist protocol.

The primary endpoint of the study was cumulative live birth rate (CLBR) in cycles with estradiol drop compared to cycles without estradiol drop. Secondary objectives included other clinical outcomes and the analysis of the potential association between cycle related characteristics and estradiol drop. Clinical outcomes analyzed were cycle cancellation rate, percentage of cycles with zero oocytes retrieved, number of oocytes retrieved, number of two-pronuclei (2PN) fertilized oocytes, utilization rate, embryo transfer, positive beta human chorionic gonadotropin (β-hCG), ongoing pregnancy and live birth rate. Cycle related characteristics included age category at the start of the cycle, type of gonadotropin, oral contraceptive pill (OCP) pretreatment, first day of GnRH antagonist administration, total dose of FSH, rank of the antagonist cycle, anti-Müllerian hormone (AMH) value, serum LH at the start of the cycle, difference in serum LH before and after first antagonist administration, serum LH on the day of ovulation triggering and serum progesterone on the day of ovulation triggering.

The study protocol was approved by the Institutional Review Board at the Antwerp University Hospital (EC 20/18/240). The study was performed according to the Strengthening the reporting of observational studies in epidemiology (STROBE) guidelines (18).

### 2.2 Stimulation protocols

The choice of protocol was made by the treating physician, based on a case-by-case evaluation of patient characteristics. An OCP was used in case of irregular cycles or for scheduling purposes.

Patients received a GnRH antagonist for pituitary suppression in order to prevent a premature LH surge. Ovarian stimulation was performed using either hMG (Menopur^®^, Ferring, Germany) or a recombinant follicle-stimulation hormone (rec-FSH α (Gonal-F^®^, Merck, Italy; Bemfola^®^, Gedeon Richter, Hungary), β (Puregon^®^, Organon, The Netherlands), δ (Rekovelle^®^, Ferring, Germany), corifollitropin α (Elonva^®^, Organon, The Netherlands) or follitropin α + lutropin α (Pergoveris^®^, Merck, Italy).

Patients underwent a baseline evaluation (ultrasound and hormonal analysis), after which they started FSH stimulation on day three of the menstrual cycle or after a washout of at least five days after discontinuation of the OCP.

The starting dose of FSH was determined based on age and serum AMH level. Patients younger than 37 years with an AMH level of 1–4 µg/L received 150 IU per day, those with AMH > 4 µg/L received 112.5 IU per day, and those with AMH <1 µg/L received 225 IU per day. All patients aged 37 years or older received 225 IU per day with AMH levels ≤ 4 µg/L or 150 IU per day with AMH > 4 µg/L.

Dosing adjustments were made according to follicular response. The GnRH antagonist was administered in a flexible protocol, meaning it was started when the estradiol level was ≥ 250 ng/L and/or ultrasound showed a follicle with a diameter of ≥14 mm. Ovulation triggering was performed with hCG or a GnRH agonist. Cycles were cancelled because of premature LH rise, poor ovarian response, Covid infection or non-medical reasons.

### 2.3 Hormone assays

Blood analysis was used to measure serum FSH, LH, hCG, estradiol and progesterone at the baseline evaluation and LH, estradiol and progesterone throughout the follicular phase of the OS. The last follicular phase measurements were performed on the day of ovulation triggering. All blood analyses were performed in the morning, immediately followed by ultrasound monitoring. LH values were analyzed using chemiluminescent 2-site sandwich immunoassay and were expressed in IU/L. Estradiol and progesterone were both analyzed using electrochemiluminescence sandwich assays and expressed in respectively ng/L and µg/L.

Serum estradiol levels were measured using the Elecsys Estradiol III immunoassay (Roche Diagnostics) with a limit of detection of 5 pg/mL. Intra-assay CV ranged from 1.2% to 2.4%, with a CV of 8.4% at the lower detection level. Inter-assay precision ranged from 1.7% to 2.7%, with a coefficient of variation (CV) of 12.3% at the lower detection level (18.7 pg/ml). Serum LH was analyzed using the Atellica IM LH chemiluminescent two-site sandwich immunoassay with an analytical sensitivity of ≤ 0.07 IU/L, an intra-assay CV between 1.1% and 1.8% and an inter-assay CV ranging from 2.1% to 3.4%.

Progesterone was measured with Elecsys Progesterone III immunoassay (Roche Diagnostics) with a limit of detection of 0.05 ng/mL. The intra-assay CV ranged from 1.1% to 2.7% and the inter-assay CV ranged from 1.7% to 6.2% with a CV of 43.9% at the lower detection level (0.054 ng/mL). AMH levels were measured with Elecsys AMH Plus (Roche Diagnostics) with a detection limit of 0.01 ng/mL, an intra-assay CV between 0.8% and 2.1% and an inter-assay CV between 2.8% and 3.8%. All hormonal assays were subjected to routine internal quality control procedures.

### 2.4 Statistical analysis

Data were extracted from medical records in two databases that were consecutively used at the Center for Reproductive Medicine. Data cleaning was performed and input errors that could be corrected with the use of reliable source data, were corrected manually. Data that could not be corrected were marked as missing. A comprehensive list of all data handling was created.

Descriptive statistics were used to describe patient and cycle characteristics. Continuous data were checked for normal distribution through statistical tests (Shapiro-Wilk test) and graphical representation (histogram and quantile-quantile plot). As data were not normally distributed, descriptive statistics were presented as median and interquartile range [q1 to q3]. Categorical variables were presented with counts and percentages.

To determine if a drop in serum estradiol levels was present, the difference between estradiol levels before and after the first GnRH antagonist administration was calculated. Ideally, the last measurement before the start of the antagonist was used, but when this value was not available, the value up to three days before was used. The value after was ideally from a blood analysis performed two days after the start of the antagonist, but values up to four days after were used when needed. When the value of estradiol after the start of the antagonist was lower than the value before, the cycle was marked as having an estradiol drop. Estradiol drop was analyzed as a binary variable.

Cumulative live birth (CLB) was defined as at least one live birth per aspirated ART cycle, according to the International Committee for Monitoring Assisted Reproductive Technologies (ICMART) definition (19). Cycles were included for analysis of CLB when a live birth had been achieved or if all embryos from that aspiration cycle had been transferred.

Due to the database containing multiple cycles per patient, the primary outcome CLBR was analyzed with a generalized estimating equations (GEE) model with subject as clustering variable (correcting for cycles of the same patient) with an exchangeable correlation structure and a logit link function. In this model there was adjusted for age class at the start of the cycle (<35y, 35-39y, ≥40y).

For the secondary binary clinical outcomes the same model was fitted as for the primary outcome. For the numeric clinical outcomes a linear mixed-effects model (including subject as a random intercept to correct for cycles of the same patient) was used with adjustment for age class at the start of the cycle.

Model assumptions were evaluated for both linear mixed-effects models and GEE models. For mixed models, residuals were visually inspected for normality, homoscedasticity, and linearity. For GEE models, QIC values guided the choice of correlation structure. Multicollinearity was explored using VIFs.

To evaluate potential confounding, several separate models were evaluated adding *a priori* selected clinically relevant covariates one by one to a base model that included estradiol drop and age at the start of the cycle. A variable was considered a confounder if its inclusion changed the β-coefficient (regression coefficient in the log odds model) of estradiol drop by more than ten percent.

As we expect an estradiol increase during OS, any decrease in estradiol was defined as drop. To challenge this arbitrary cutoff, a sensitivity analysis was performed for the main analysis defining estradiol drop as all decreases or increases not exceeding five percent (binary outcome named “expanded estradiol drop”).

Additionally, to further explore the impact of ranges of estradiol drop on CLBR, a second sensitivity analysis was performed categorizing the percent change in estradiol (no drop, 0-10% drop, 10-20% drop and > 20% drop). To visualize the association between percentage change in estradiol levels after GnRH antagonist initiation and CLBR, predicted probabilities for different age categories were derived from the GEE model using the logit link. The probabilities were plotted with 95% confidence intervals (CI’s) across a range of a drop of 100% to an increase of 10%.

To analyze whether patient and cycle characteristics (explanatory variables) were associated with the occurrence of an estradiol drop GEE models were used using estradiol drop as outcome and subject as clustering variable with an exchangeable correlation structure and a logit link function.

We visualized estradiol and LH evolution throughout the follicular phase of OS by plotting the mean estradiol (ng/L) or LH value (IU/L) against the day of GnRH antagonist administration, with 95% CI’s. Day 1 on the x-axis represents the first day of GnRH antagonist administration and estradiol/LH was plotted from day ten before start of antagonist until day ten of administration. CI’s were plotted when there were at least three measurements at a certain time point. Linear mixed-effects models (using subject as random effect) with and without interaction between cycle day and estradiol drop were compared to assess whether estradiol profiles differed between cycles with and without estradiol drop.

To evaluate whether the impact of estradiol drop could be visualized by ultrasound monitoring, follicle measurements were analyzed. The mean diameter of the same number of leading follicles right before and right after the first administration of the GnRH antagonist (the same days as used to calculate estradiol drop) was calculated for cycles with estradiol drop.

Statistical analysis was performed using R Studio version 4.3.0. P-values of less than 0.05 indicated statistical significance.

### 2.5 Missing data handling

For use of OCP pre-treatment and serum AMH value substantial percentages of missing data were detected (38.9% and 47.7% respectively). Therefore, missing data for both were imputed using multiple imputations under the assumption of data missing at random. Multiple imputation was performed using the Multivariate Imputation by Chained Equations package in R. In the imputation method the variables OCP, AMH, age at the start of the cycle, type of treatment, type of gonadotropin, total dose of gonadotropins, start day of the GnRH antagonist, rank of the cycle, LH level at the start of the cycle, LH and progesterone levels at ovulation triggering, date of oocyte retrieval and presence of estradiol drop were used. These variables were selected as they could be associated with OCP use and AMH levels. Subject and cycle identifiers were included to allow for clustering.

Predictive mean matching was used for continuous variables, logistic regression for binary variables and polytomous regression for categorical variables with more than two levels. Twenty imputation datasets were generated using five iterations per dataset. Distributions of the variables before and after imputation are presented in **Supplementary Table 1**.

Two models were fitted with each imputed dataset: a GEE model with estradiol drop as outcome and AMH as independent variable and a GEE model with estradiol drop as outcome and OCP independent variable. An exchangeable working correlation matrix was specified to account for within-subject correlation across cycles (clustered by subject identifier). Pooled parameter estimates from twenty imputations were calculated using Rubin’s rules.

To assess potential confounders in the association between estradiol drop and CLBR, additional GEE models were fitted in the imputed datasets. An exchangeable working correlation matrix was again specified to account for within-subject correlation. Pooled parameter estimates were obtained across the twenty imputed datasets using Rubin’s rules.

### 2.6 Sample size

This study used a sample of convenience, including all patients treated with a GnRH antagonist for IVF/ICSI in our center between January 1st 2015 and November 1st 2022.

## 3 Results

### 3.1 Patient and cycle characteristics

**Figure 1:**
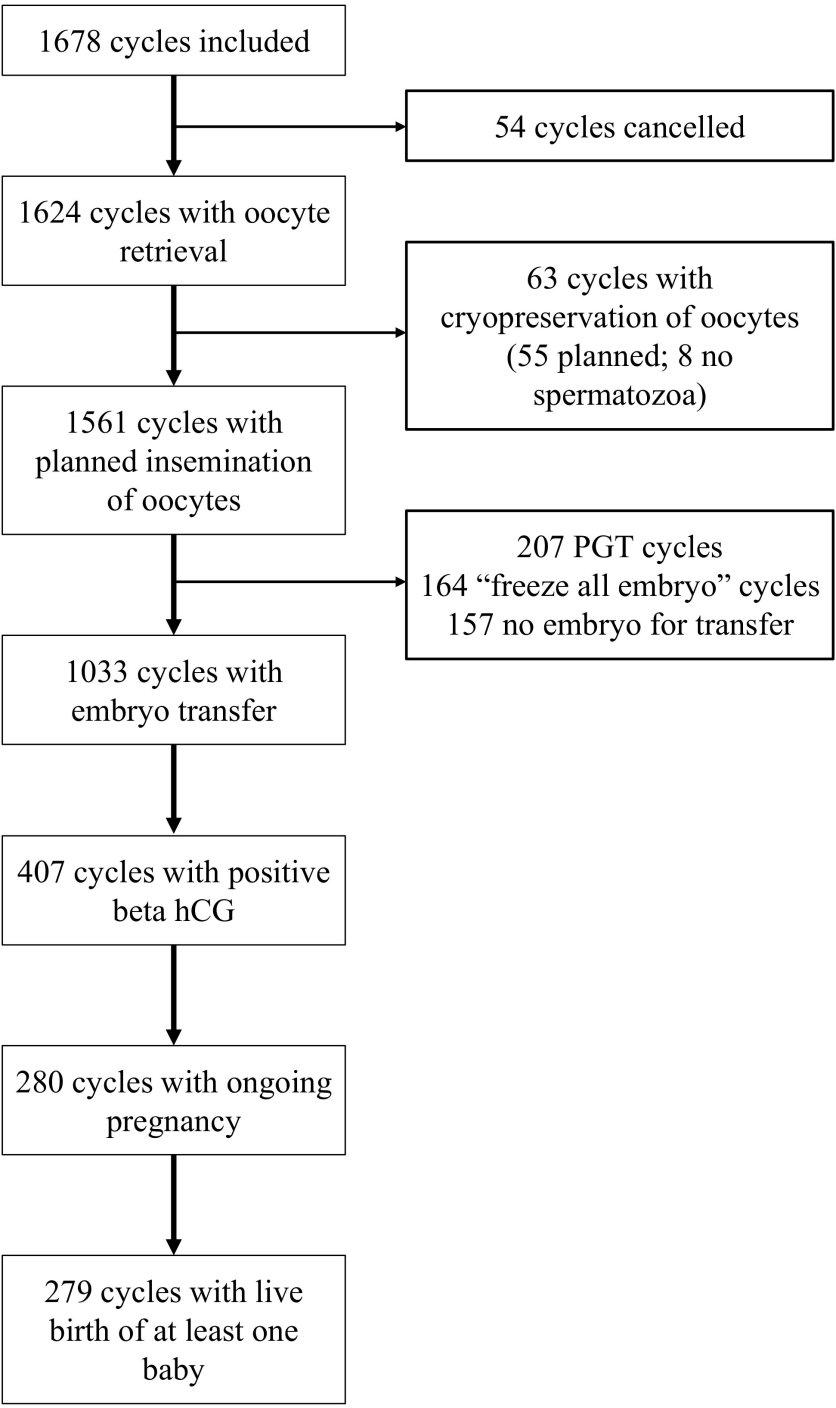
Flow diagram of cycles through treatment steps.

We analyzed 1678 cycles in 1143 unique patients, as described in the flow diagram in **Figure 1**. For the analysis of CLBR 1331 cycles could be included. Cycle characteristics are listed in **Table 1**.

**Table 1:** Cycle characteristics and association with estradiol drop (univariable analysis).

Cycle characteristic	*Overall*	*Cycles without E_2_ drop*	*Cycles with E_2_ drop*	*p-value*
Age at the start of the cycle	33 [30 – 37]	33 [30 – 36]	33 [30 – 38]	0.67
Age category at the start of the cycle				0.73
< 35y	1046 (62.3)	885 (63.3)	94 (61.4)	
35 – 39y	418 (24.9)	347 (24.8)	38 (24.8)	
≥ 40y	214 (12.8)	167 (11.9)	21 (13.7)	
*Total*	*1678*	*1399*	*153*	
Rank of the antagonist cycle (until live birth)	1 [1 - 2]	1 [1 - 2]	1 [1 - 2]	0.09
Type of treatment cycle				0.53
IVF	645 (38.4)	535 (38.2)	55 (35.9)	
ICSI	640 (38.1)	530 (37.9)	66 (43.1)	
IVF + ICSI	124 (7.4)	102 (7.3)	8 (5.2)	
PGT	214 (12.8)	183 (13.1)	23 (15.0)	
cryopreservation of oocytes	55 (3.3)	49 (3.5)	1 (0.7)	
*Total*	*1678*	*1399*	*153*	
Use of OCP pre-treatment				0.23 ^1^
yes	211 (20.6)	186 (21.3)	9 (10.11)	
no	814 (79.4)	687 (78.7)	80 (89.9)	
*Total*	*1025 ^2^*	*873*	*89*	
Type of FSH				*<0.0001*
hMG	708 (42.2)	623 (44.5)	19 (12.4)	
follitropine α/β	417 (24.9)	338 (24.2)	50 (32.7)	
follitropine δ	80 (4.8)	72 (5.1)	6 (3.9)	
corifollitropin α (+hMG/rec-FSH)	388 (23.1)	292 (20.9)	74 (48.4)	
follitropine α + lutropine α	85 (5.1)	74 (5.3)	4 (2.6)	
*Total*	*1678*	*1399*	*153*	
Total dose of FSH (IU)	1688 [1350 – 2215]	1688 [1350 – 2215]	1800 [1540 – 2270]	0.24
First day of GnRH antagonist administration				0.32
day 6 of stimulation	1025 (61.1)	861 (61.5)	99 (64.7)	
later than day 6 of stimulation	653 (38.9)	538 (38.5)	54 (35.3)	
*Total*	*1678*	*1399*	*153*	
Serum estradiol before antagonist initiation (ng/L)	533 [321 - 825]	526 [321 – 803]	627 [333 – 1079]	*<0.0001*
Serum estradiol after antagonist initiation (ng/L)	936 [604 – 1383]	981 [666 – 1441]	475 [225 – 853]	*<0.0001*
Serum LH at the start of the cycle (IU/L)	3.50 [1.80, 5.30]	3.50 [1.80 – 5.30]	3.60 [1.80 – 5.10]	0.26
Difference in serum LH before and after start of GnRH antagonist (IU/L)	-1.00 [-2.30 - -0.30]	-0.90 [-2.10 - -0.30]	-2.40 [-5.95 - -0.90]	*<0.0001*
Serum LH on day of triggering (IU/L)	1.10 [0.50 – 2.20]	1.20 [0.60 – 2.30]	0.70 [0.20 – 1.50]	*0.03*
Serum progesterone on day of triggering (µg/L)	0.43 [0.25 – 0.70]	0.43 [0.25 – 0.69]	0.40 [0.24 – 0.82]	0.32
AMH at start of treatment (µg/L)	1.89 [0.87 – 3.66]	1.98 [0.92 – 3.72]	1.53 [0.49 – 2.52]	0.82 ^1^

Continuous variables are reported as median [IQR], categorical variables are reported as n (%).

^1^p-value derived from pooled analyses across the imputed datasets (20 imputations).

^2^Discrepancy in sample size due to non-systematic recording in early study years.

**Figure 2:**
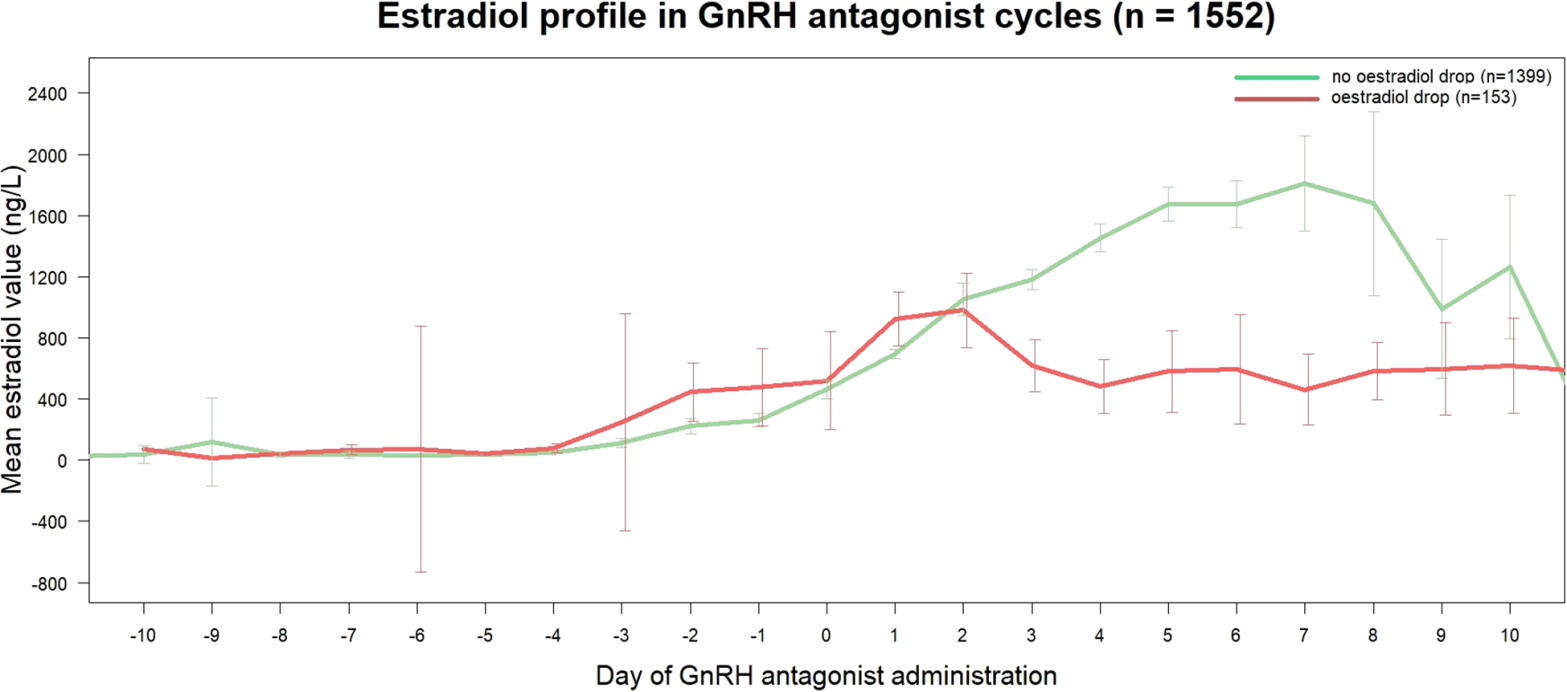
Estradiol profiles in GnRH antagonist cycles according to presence of estradiol drop. Data are expressed as mean ± 95% CI.

The median number of days between measures of estradiol levels before and after the first GnRH antagonist administration was 2 [2 to 3]. The presence of estradiol drop could be calculated in 1552 cycles, of which 153 cycles (9.9%) showed a drop. The median decrease in estradiol levels in these drop cycles was -122 [-247 to -39] ng/L, which translates to a mean decrease of -21.72 [-36.93 to -8.99] %. The expanded estradiol drop was found in 181 cycles (11.7%), with a median decrease in estradiol levels of -71 [-223 to -16] ng/L. This was a median decrease of -16.02 [-33.55 to -3.31] %. **Figure 2** shows the estradiol evolution throughout the follicular phase of ovarian stimulation. Estradiol profiles were statistically different over time between cycles with and without estradiol drop (p < 0.0001).

### 3.2 Association between estradiol drop and clinical outcomes

The percentage of cancelled cycles was significantly higher in the estradiol drop group (5.9%) than in the group without estradiol drop (1.9%, OR 3.30, 95% CI [1.46, 7.45], p = 0.004) as well as the number of cycles in which zero oocytes were retrieved estradiol (4.2% versus 1.0%, OR 4.08, 95% CI [1.48, 11.25], p = 0.007). However, no association was found for the mean number of oocytes retrieved, mean number of 2PN fertilized oocytes and utilization rate.

**Figure 3:**
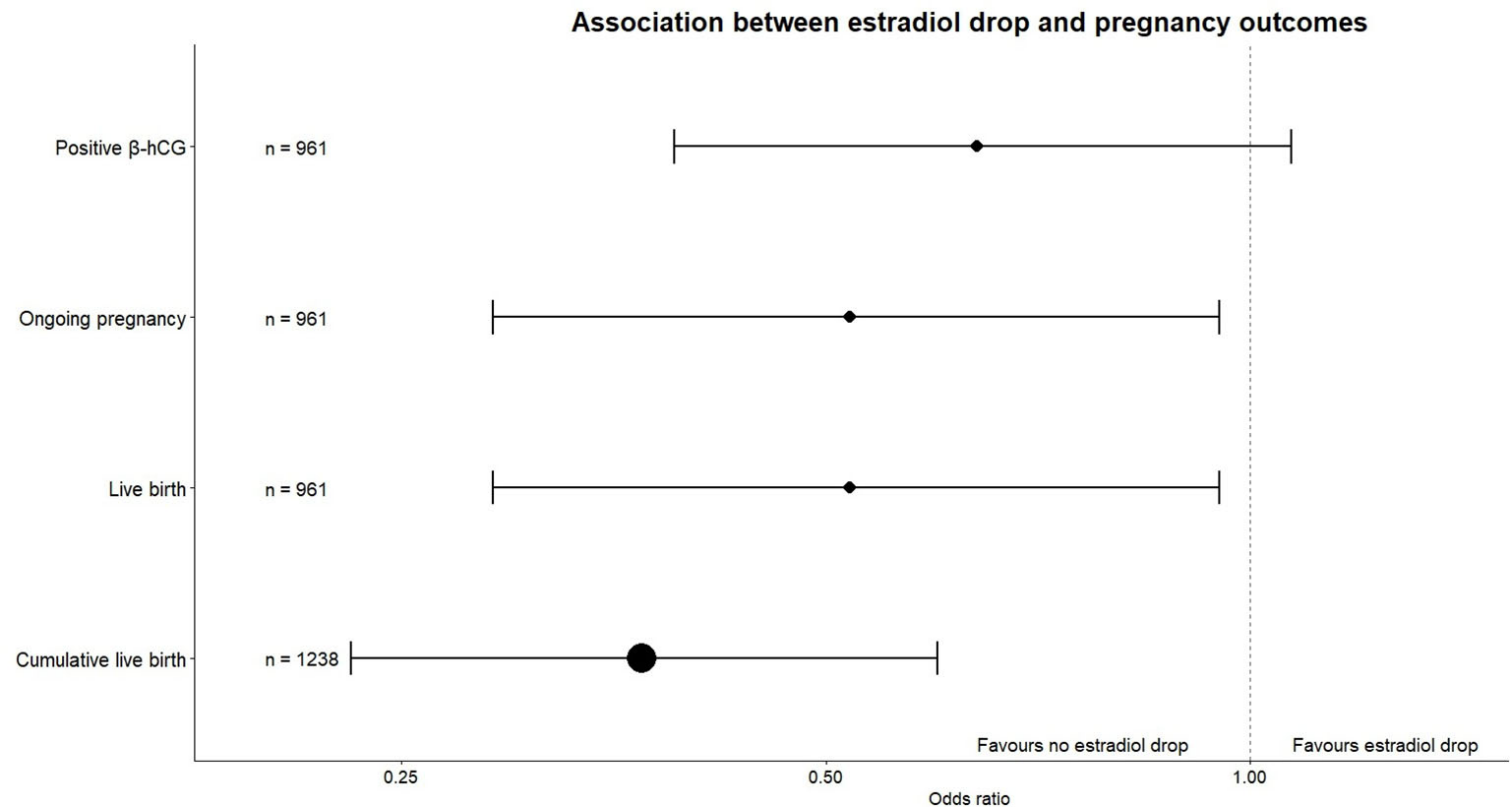
Forest plot of association between estradiol drop and pregnancy outcomes.

The percentage of cycles in which at least one embryo was transferred in the fresh cycle was significantly lower in the cycles with estradiol drop (69.2% versus 77.2%, OR 0.64, 95% CI [0.42, 0.98], p = 0.04). Ongoing pregnancy rates were significantly lower in estradiol drop cycles (16.9%) than in cycles without estradiol drop (28.0%, OR 0.52, 95% CI [0.29, 0.95], p = 0.03). The percentage of cycles with a live birth also differed significantly (27.9% versus 16.9%, OR 0.52, 95% CI [0.29, 0.95], p = 0.03). Cumulative live birth rate was significantly lower in the estradiol drop group (20.0%) compared to the group without drop (38.7%, OR 0.37, 95% CI [0.23, 0.60], p < 0.0001) (**Table 2**, **Figure 3**).

**Table 2:** Association between estradiol drop and clinical outcomes.

Variable	Total N cycles ^5^	No E_2_ drop	E_2_ drop	p-value ^6^	*OR [95% CI]*	*diff. in LS means [95% CI] ^7^*
Cycles cancelled (n (%))	1552	26 (1.9)	9 (5.9)	0.004	3.30 [1.46,7.45]	
Cycles with zero oocytes retrieved (n(%)) ^1^	1517	14 (1.0)	6 (4.2)	0.007	4.08 [1.48, 11.25]	
Number of oocytes retrieved (LS mean ± SE) ^1^	1517	8.26 ± 0.22	7.66 ± 0.49	0.22		0.59 [-0.36, 1.54]
Number of 2PN fertilized (LS mean ± SE) ²	1461	4.76 ± 0.16	4.21 ± 0.35	0.12		0.55 [-0.14, 1.24]
Utilization rate (LS mean ± SE (%)) ³	1258	46.9 ± 1.16	42.3 ± 3.11	0.15		4.64 [-1.63, 10.9]
Embryo transfer (n (%)) ³	1258	878 (77.2)	83 (69.2)	0.04	0.64 [0.42, 0.98]	
Positive β-hCG (n (%)) ^4^	961	352 (40.1)	25 (30.1)	0.09	0.64 [0.39, 1.07]	
Ongoing pregnancy (n (%)) ^4^	961	246 (28.0)	14 (16.9)	0.03	0.52 [0.29, 0.95]	
Live birth (n (%)) ^4^	961	245 (27.9)	14 (16.9)	0.03	0.52 [0.29, 0.95]	
Cumulative live birth rate ³ (%)	1238	38.7	20.0	< 0.0001	0.37 [0.23, 0.60]	

^1^Calculated on all cycles with oocyte retrieval.

²Calculated on all cycles with oocyte retrieval, excluding cryopreservation of oocytes.

³Calculated on all cycles with oocyte retrieval, excluding cryopreservation of oocytes and PGT.

^4^Calculated on all cycles with embryo transfer.

^5^Number of cycles for which presence of estradiol drop could be calculated.

^6^Adjusted for age at the start of the cycle.

^7^Applicable only for numeric outcomes.

When adjusting for potential confounders, the association between estradiol drop and CLBR remained highly significant across all models. The β-coefficients after adjustments for individual covariates remain comparable and the 95% CIs show a similar range of clinical meaningful differences (**Supplementary Table 2**).

### 3.3 Sensitivity analyses

When analyzing the association between the expanded estradiol drop and clinical outcomes, the percentage of cancelled cycles, as well as the percentage of cycles with zero oocytes retrieved remained significantly higher (respective p-values = 0.0002 and 0.02). CLBR remained significantly lower in cycles with estradiol drop (23.1% versus 38.7% in cycles without estradiol drop, OR 0.45, 95% CI [0.29, 0.69], p < 0.0001). All outcomes analyzed with the expanded estradiol drop are shown in **Supplementary Table 3**.

In a sensitivity analysis categorizing the percent change in estradiol (no drop, 0–10%, 10–20%, and >20%), this estradiol difference remained significantly associated with CLBR (p = 0.003). Using cycles without estradiol drop (n = 1138 (90.5%)) as a reference, declines exceeding 10% were associated with significantly lower odds of cumulative live birth (10–20% (n = 32 (2.5%)): OR 0.28, 95% CI [0.11, 0.72]; >20% (n = 56 (4.5%)): OR 0.59, 95% CI [0.39, 0.88]). The 0–10% category (n = 32 (2.5%)) was not significantly associated with CLBR (OR 0.57, 95% CI [0.26, 1.23]).

Predicted probabilities of cumulative live birth decreased progressively with increasing estradiol drop, with consistently lower probabilities observed in older age groups (**Supplementary Figure 1**).

### 3.4 Association between treatment and cycle characteristics and estradiol drop

All variables that were analyzed for possible association with estradiol drop are listed in **Table 1**.

Estradiol drop appeared to be significantly associated with the different types of gonadotropin (p < 0.0001). Using follitropin α or β as a reference, both hMG and follitropin α + lutropin α were associated with significantly lower odds of estradiol drop (hMG: OR 0.22, 95% CI [0.13, 0.39], p < 0.0001; follitropin α + lutropin α: OR 0.33, 95% CI [0.11, 0.97], p = 0.04). In contrast, corifollitropin α showed significantly higher odds (OR 1.99, 95% CI [1.32, 2.99], p = 0.001), while no statistically significant difference was observed for follitropin δ (OR 0.62, 95% CI [0.24, 1.62], p = 0.33).

**Figure 4:**
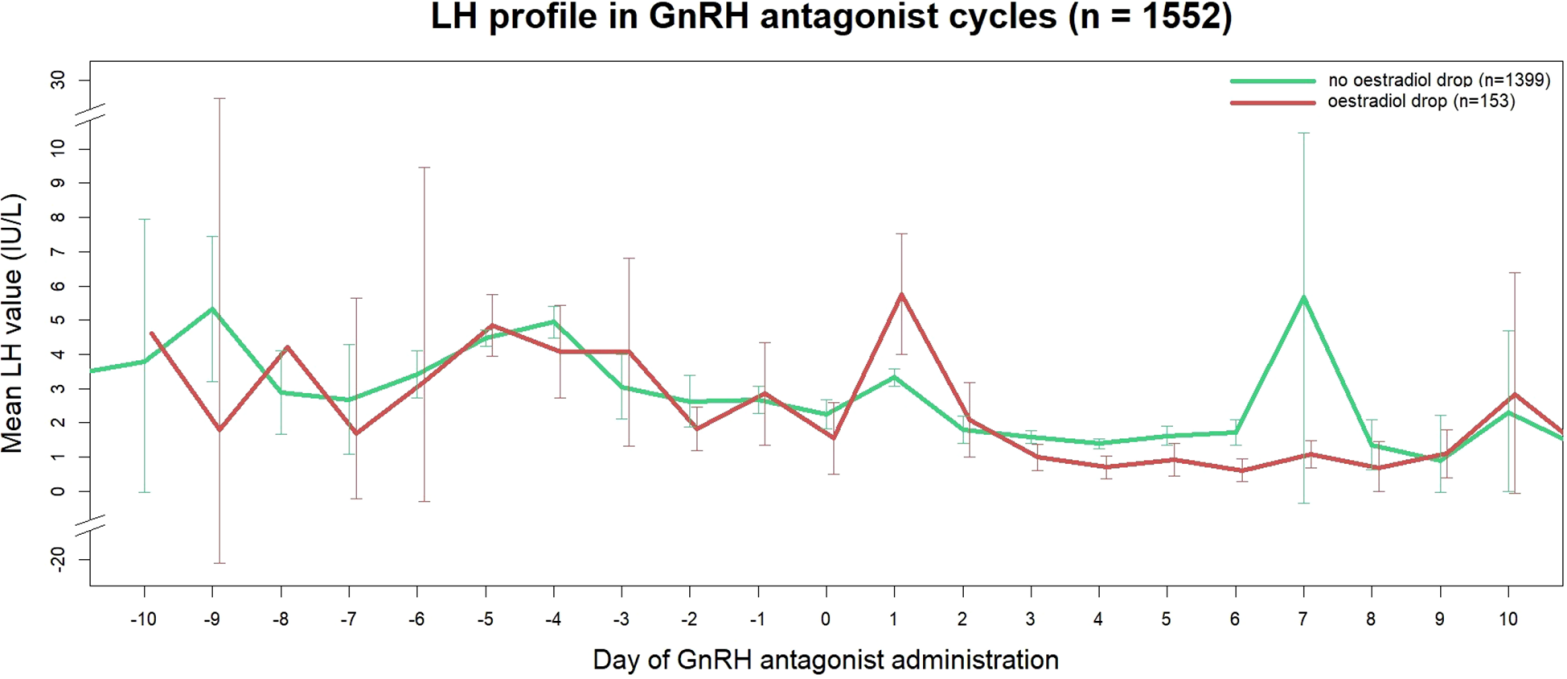
LH profiles in GnRH antagonist cycles according to presence of estradiol drop. Data are expressed as mean ± 95% CI.

Serum LH levels seemed to be associated with estradiol drop. Cycles exhibiting an estradiol drop showed a larger decrease in LH after the first GnRH antagonist administration (-2.40 [-5.95 to -0.90] IU/L, versus -0.90 [-2.10 to -0.30] in cycles without drop, p < 0.0001), resulting in lower serum LH values on the day of ovulation triggering (0.70 [0.20 to 1.50] IU/L, versus 1.20 [0.60 to 2.30] IU/L in cycles without drop, p = 0.03). **Figure 4** shows the evolution of serum LH throughout ovarian stimulation for cycles with and without estradiol drop.

### 3.5 Follicle measurements in estradiol drop

The mean follicle diameter of the leading group of follicles was compared before and after the first GnRH antagonist administration in 148 of 153 cycles with estradiol drop. Of these cycles, 14 (9.5%) showed a stagnation of follicle growth after antagonist initiation.

## 4 Discussion

In our study, 9.9% of cycles showed an estradiol drop after the first GnRH antagonist administration. Significantly more cycles were cancelled in the estradiol drop group and the number of cycles in which no oocytes were retrieved was also significantly higher. For the cycles in which an oocyte retrieval was performed, ongoing pregnancy, live birth and cumulative live birth rates were all significantly lower in cycles that presented with an estradiol drop. To our knowledge, these findings have not been published yet in existing literature.

Although no statistically significant associations were observed for the number of oocytes retrieved, number of 2PN fertilized oocytes, utilization rate and positive β-hCG, the direction of the effect of the estimates was consistent with the observed reduction in CLBR. While the confidence intervals were largely aligned with the hypothesized direction, their width reflects limited precision and clinically relevant effects of a smaller magnitude, or even no effect cannot be excluded.

### 4.1 Factors associated with estradiol drop

With respect to cycle specific characteristics, the type of gonadotropin used for OS was associated with the presence of an estradiol drop. Significantly lower percentages of estradiol drop cycles were detected in cycles stimulated with hMG and follitropin α + lutropin α, both of which contain a form of LH activity (20).

Serum LH measurements were associated with estradiol drop. Although there was no significant association between serum LH at the start of the cycle, the decrease in LH after first antagonist administration was significantly larger in cycles with estradiol drop, resulting in a lower serum LH value on the day of ovulation triggering.

The association between suppressed LH and impaired estradiol synthesis may be explained by the two-cell, two-gonadotropin system. In the early follicular phase, LH receptors are primarily expressed in theca cells, where LH stimulates the conversion of cholesterol into androgens. These androgens subsequently diffuse to granulosa cells, in which FSH promotes their aromatization into estrogens. Suppression of LH may therefore reduce theca cell androgen output, limiting substrate availability for aromatization and consequently impairing estradiol synthesis.

In the late follicular phase FSH induces the expression of LH receptors in granulosa cells, allowing LH to augment FSH-mediated steroidogenesis and thereby enhance estradiol production (21). By acquiring LH responsiveness, the maturing follicle becomes increasingly dependent on adequate LH support (22–24). Excessive LH suppression during this stage may therefore compromise optimal steroidogenic activity, follicular maturation and potentially oocyte competence. Our previous research showed a significant decrease in serum LH in GnRH antagonist cycles, which was significantly more pronounced in cycles were recombinant FSH was used than in cycles with hMG (Janssens et al., 2022). In that study we did not detect a decrease in LH in GnRH agonist long cycles, suggesting this may be of benefit for the patients that suffer from estradiol drop. The role of LH in ovarian stimulation has been a longstanding topic of discussion in literature and yet no ideal range of serum levels has been defined. Many studies have been performed comparing FSH preparations with and without LH activity in terms of follicular response and clinical outcomes (25–27). A meta-analysis found that FSH alone might result in a higher number of oocytes, but added LH activity improved oocyte and embryo quality (28). Exogenous LH may partially counteract antagonist-induced suppression of endogenous LH. By restoring theca cell androgen production and maintaining adequate substrate availability for aromatization, LH supplementation may preserve intrafollicular steroidogenesis and thereby support oocyte competence. This mechanism could explain why LH-containing gonadotropins appear to mitigate the negative impact of profound LH suppression induced by the GnRH antagonist on clinical outcomes.

The benefit of LH supplementation during OS in several specific patient populations has been demonstrated by a growing number of studies, but no consensus exists on its universal application (29). Furthermore, variations in LH bioactivity highlight the significance of a precise case-by-case refinement of serum LH levels in order to obtain optimal clinical outcomes (29–31).

### 4.2 How to handle estradiol drop?

Significantly more cycles with estradiol drop were cancelled before oocyte retrieval and in cycles with oocyte retrieval, the incidence of retrieving no oocytes was significantly higher. The mean number of oocytes retrieved, number of 2PN fertilized oocytes and the utilization rate were not significantly different between both groups. So how do we handle this drop and should we cancel the cycle?

As Quaas et al. stated, ART is very result-driven and both patient and clinician may anticipate that changing the stimulation protocol will lead to a better outcome (3). From our research we may speculate that the use of gonadotropins with LH activity or a long agonist protocol might protect patients from estradiol drop and related poorer clinical outcomes. Nevertheless, we might be overestimating the effect of the protocol and simply giving the patient another chance might be more important than the protocol itself (3). The decision to cancel or continue the cycle might also be influenced by the reimbursement policy. The risk of losing a reimbursed attempt to a cycle with poor outcomes might add to the choice to cancel the cycle.

### 4.3 Do we need to measure serum estradiol?

Regarding estradiol measurements, our findings might add to the discussion that estradiol monitoring could be useful in detecting cycles with an increased risk of poor outcome given the observation that in cycles with an estradiol drop a stagnation of follicle growth was only detected by ultrasound monitoring in 9.5% of the drop cycles. Estradiol secretion by the granulosa cells of these follicles could already be impaired (32), without it being detectable by ultrasound tracking and serum estradiol measurements might be a more sensitive method to identify compromised cycles.

### 4.4 Limitations and wider implications

Given the retrospective design, we were unable to correct for all possible confounders in one model. However, adjustment for repeated cycles per patient was performed using linear mixed models and generalized estimating equations models. Because absolute numbers of estradiol drop were low this caused a limitation to perform an optimal multivariable analysis and to confirm a statistically valid threshold for percentage of estradiol drop. In addition, residual confounding by unmeasured factors such as body mass index or infertility diagnosis cannot be excluded.

While the median time window between estradiol measurements was narrow (2 [2-3] days), the timing was not standardized across cycles. A 3-day offset prior to GnRH antagonist initiation was required only in a very small proportion of cycles and none of these were classified as having an estradiol drop. The vast majority of cycles with a drop had estradiol measured on the day of antagonist initiation. Therefore, it is unlikely that minor variability in timing influenced cycle classification. While small differences in the exact magnitude of the estradiol change cannot be entirely excluded, the presence of an estradiol drop itself represents a clinically relevant observation during ovarian stimulation.

Although the study was retrospective and used a convenience sample, the effect estimate of 0.37 shows about 63% lower odds on CLBR in cycles with estradiol drop compared to cycles without estradiol drop with the 95% confidence interval allowing the true effect to range of 40% to 77% lower odds, which regardless of the precise value, represents a clinical important difference. We acknowledge that a prospective study with an *a priori* sample size calculation, standardized methodology and appropriate adjustment for potential confounders is needed to validate and generalize study findings.

No consensus exists on the added value of estradiol measurements in addition to ultrasound monitoring during ovarian stimulation. The findings of this study might add to the discussion that estradiol monitoring could be useful in detecting cycles with an increased risk of less favorable outcomes. It should be acknowledged that ultrasound assessment is inherently operator-dependent and subject to some degree of intra- and interobserver variability (17, 33). In this study, ultrasound monitoring was performed by the same team of senior clinicians following standardized protocols, which likely limited measurement variability. An analysis of the combined predictive value of ultrasound monitoring and estradiol measurements may further clarify their complementary roles in optimizing ovarian stimulation strategies.In conclusion, estradiol drop after GnRH antagonist administration was associated with lower cumulative live birth rates. Regarding treatment and cycle characteristics, estradiol drop was associated with type of gonadotropin and changes in luteinizing hormone. Prospective research could focus on potential optimization of LH levels, which could possibly ameliorate clinical outcomes.

## Data Availability

The datasets presented in this study can be found in online repositories. The names of the repository/repositories and accession number(s) can be found below: Figshare DOI 10.6084/m9.figshare.30307567.
