# Supplementary figures and images for: A drop in serum estradiol levels during GnRH antagonist cotreatment in cycles stimulated with gonadotropins is associated with lower cumulative live birth rates
Source: Front Endocrinol (Lausanne). 2026 Mar 18;17:1722379. doi: 10.3389/fendo.2026.1722379 (PMC13038525; doi:10.3389/fendo.2026.1722379)

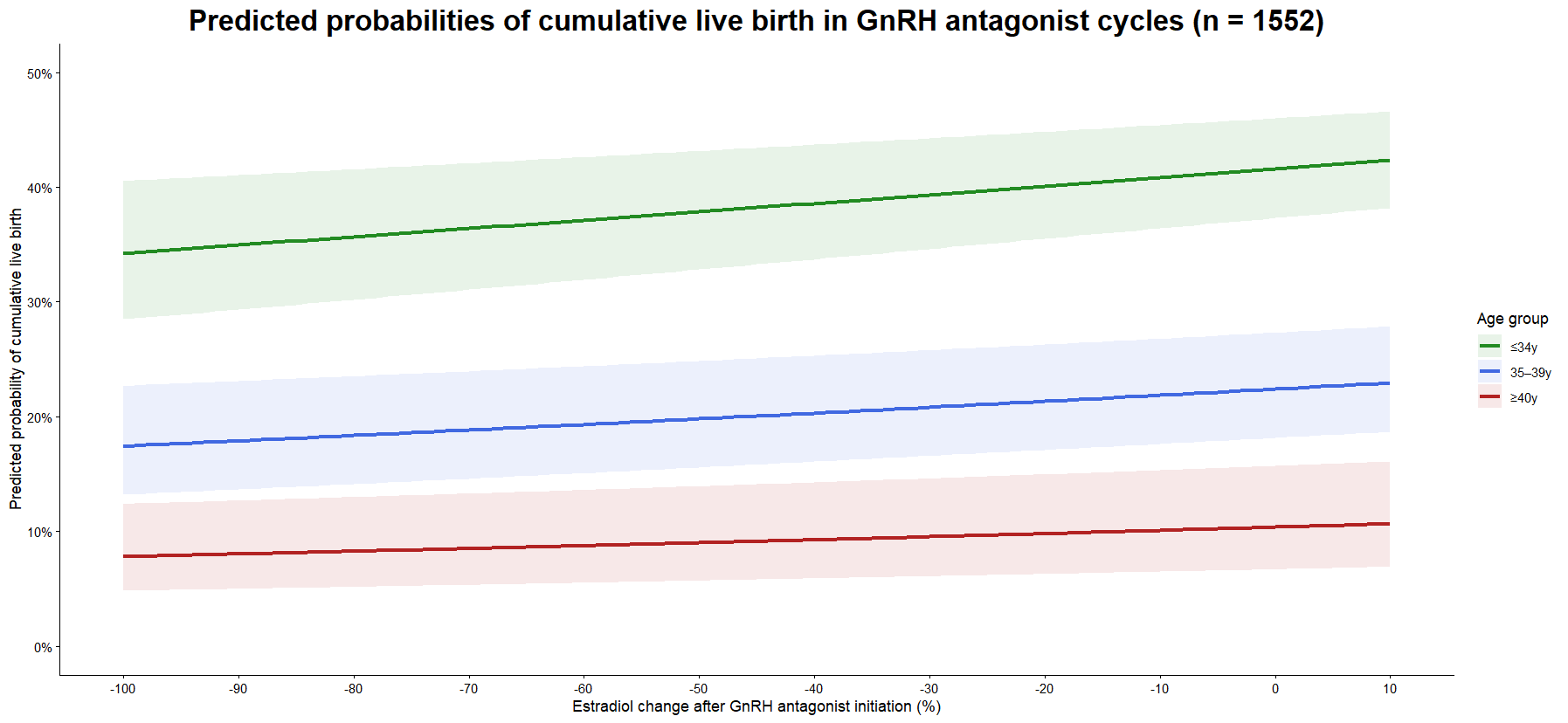

Supplement: Supplementary file 1 [file Image1.png]
