# Supplementary material for: A drop in serum estradiol levels during GnRH antagonist cotreatment in cycles stimulated with gonadotropins is associated with lower cumulative live birth rates
Source: Front Endocrinol (Lausanne). 2026 Mar 18;17:1722379. doi: 10.3389/fendo.2026.1722379 (PMC13038525; doi:10.3389/fendo.2026.1722379)
Supplement: Supplementary file 2 [file Table1.docx]

| **Table S1. Distributions of variables before and after imputation** | | | |
| --- | --- | --- | --- |
| ***Variable*** | | ***Before imputation*** | ***After imputation*** |
| Use of OCP pre-treatment (n (%)) | |  |  |
|  | yes | 211 (20.6) | 539 (31.2) |
|  | no | 814 (79.4) | 1139 (68.8) |
| AMH at the start of treatment (µg/L) | |  |  |
|  | min – max | 0.01 – 26.70 | 0.01 – 26.70 |
|  | mean | 2.83 | 3.01 |
|  | median [IQR] | 1.91 [0.90 - 3.68] | 2.19 [1.03 - 4.17] |
