# Supplementary material for: A drop in serum estradiol levels during GnRH antagonist cotreatment in cycles stimulated with gonadotropins is associated with lower cumulative live birth rates
Source: Front Endocrinol (Lausanne). 2026 Mar 18;17:1722379. doi: 10.3389/fendo.2026.1722379 (PMC13038525; doi:10.3389/fendo.2026.1722379)
Supplement: Supplementary file 3 [file Table2.docx]

| **Table S2. Confounder analysis: impact of additional covariates on the association between estradiol drop and CLBR** | | | |
| --- | --- | --- | --- |
| ***Model*** | ***Included variables in crude model*** | ***Β [95% CI]*** | ***p-value*** |
| 1 | estradiol drop + age category | -0.96 [-1.43, -0.48] | <0.0001 |
|  | ***Added variables in adjusted models*** |  |  |
| 2 | … + type of gonadotropin | -0.86 [-1.35, -0.37] | <0.001 |
| 3 | … + first day of GnRH administration | -0.95 [-1.43, -0.48] | 0.0001 |
| 4 | … + OCP pretreatment ^1^ | -0.96 [-1.43, -0.48] | <0.001 |
| 5 | … + total dose of FSH | -0.95 [-1.43, -0.47] | 0.0001 |
| 6 | … + rank of the cycle | -0.96 [-1.43, -0.48] | <0.001 |
| 7 | … + AMH value ^1^ | -0.98 [-1.46, -0.50] | <0.001 |
| 8 | … + serum LH at the start of the cycle | -0.98 [-1.46, -0.50] | <0.001 |
| 9 | … + difference in serum LH before and after antagonist | -0.92 [-1.41, -0.43] | <0.001 |
| 10 | … + serum LH on day of triggering | -0.98 [-1.45, -0.50] | <0.0001 |
| 11 | … + serum progesterone on day of triggering | -0.95 [-1.43, -0.48] | <0.0001 |

^1^ values derived from pooled analysis across the imputed datasets (20 imputations) using MICE
