# Supplementary material for: A drop in serum estradiol levels during GnRH antagonist cotreatment in cycles stimulated with gonadotropins is associated with lower cumulative live birth rates
Source: Front Endocrinol (Lausanne). 2026 Mar 18;17:1722379. doi: 10.3389/fendo.2026.1722379 (PMC13038525; doi:10.3389/fendo.2026.1722379)
Supplement: Supplementary file 4 [file Table3.docx]

| **Table S3. Association between expanded estradiol drop and clinical outcomes** | | | | |  |  |
| --- | --- | --- | --- | --- | --- | --- |
| **Variable** | **Total N cycles ^5^** | **No E_2_ drop** | **E_2_ drop** | **p-value** **^6^** | ***OR [95% CI]*** | ***diff. in LS means [95% CI] ^7^*** |
| Cycles cancelled (n (%)) | 1552 | 23 (1.7) | 12 (6.6) | 0.0002 | 4.19 [1.99, 8.85] |  |
| Cycles with zero oocytes retrieved (n(%)) ^1^ | 1517 | 14 (1.0) | 6 (3.6) | 0.02 | 3.34 [1.22, 9.20] |  |
| Number of oocytes retrieved (LS mean ± SE) ^1^ | 1517 | 8.28 ± 0.22 | 7.57 ± 0.45 | 0.12 |  | 0.71 [-0.19, 1.60] |
| Number of 2PN fertilized (LS mean ± SE) ² | 1461 | 4.77 ± 0.16 | 4.19 ± 0.33 | 0.08 |  | 0.58 [-0.07, 1.23] |
| Utilization rate (LS mean ± SE (%)) ³ | 1258 | 47.1 ± 1.17 | 41.9 ± 2.86 | 0.09 |  | 5.17 [-0.63, 11.0] |
| Embryo transfer (n (%)) ³ | 1258 | 862 (77.3) | 99 (69.2) | 0.03 | 0.64 [0.44, 0.95] |  |
| Positive β-hCG (n (%)) ^4^ | 961 | 345 (40.0) | 32 (32.3) | 0.14 | 0.70 [0.44, 1.12] |  |
| Ongoing pregnancy (n (%)) ^4^ | 961 | 241 (28.0) | 19 (19.2) | 0.06 | 0.59 [0.35, 1.02] |  |
| Live birth (n (%)) ^4^ | 961 | 240 (27.8) | 19 (19.2) | 0.06 | 0.60 [0.35, 1.03] |  |
| Cumulative live birth rate (%) ³ | 1238 | 38.7 | 23.1 | < 0.0001 | 0.45 [0.29, 0.69] |  |
| ^1^ calculated on all cycles with oocyte retrieval  ² calculated on all cycles with oocyte retrieval, excluding cryopreservation of oocytes  ³ calculated on all cycles with oocyte retrieval, excluding cryopreservation of oocytes and PGT  ^4^ calculated on all cycles with embryo transfer  ^5^ number of cycles for which presence of estradiol drop could be calculated ^6^ adjusted for age at the start of the cycle  ^7^ applicable only for numeric outcomes | | | | |  |  |
